# Hippocampal engrams configure prefrontal context representations to guide flexible decisions

**DOI:** 10.64898/2026.07.06.732916

**Published:** 2026-07-07

**Authors:** Joshua B. Julian, Jesse C. Kaminsky, David W. Tank, Carlos Brody

**Affiliations:** 1 Princeton Neuroscience Institute, Princeton, NJ, USA; 2 Howard Hughes Medical Institute, Princeton University, Princeton NJ, USA

## Abstract

Flexible behavior requires using past experiences to configure cortical computations to suit current task demands. A central question in neuroscience is how memory representations control such reconfigurations. Although hippocampal (HPC) engrams can drive learned behaviors, prior studies have been largely limited to a fixed stimulus-response computation. Thus, whether engrams can drive retrieval of a set of stimulus-response mappings, rather than a specific response itself, remains unresolved. Moreover, how engrams affect cortical task representations and dynamics so as to produce engram-consistent behavior remains unstudied. Here, we address both questions by combining tagging and reactivation of HPC engrams with simultaneous large-scale recordings in medial prefrontal cortex (mPFC) during a context-dependent task-switching paradigm in mice. We report that HPC engram reactivation caused mice to apply engram-consistent decision-rules rather than a specific motor output. Simultaneous mPFC recordings revealed that reactivating the HPC engram for a given context reinstated the representation of that context in mPFC within hundreds of milliseconds, indicating that it was mediated by rapid network effects, leading to choice behavior consistent with the reactivated HPC engram. Other aspects of endogenous dynamics in mPFC were left remarkably intact. Together, our findings provide direct causal evidence that HPC engrams can configure task-relevant population states of downstream cortical circuits in real time, establishing a neural mechanism by which memory traces control flexible behavior.

Flexible behavior depends on using context to select the appropriate set of actions that lead towards achieving one’s goals. This requires each context to engage a distinct configuration of cognitive processes, or ‘task set’, often operationalized in task-switching studies as the context-specific rules linking sensory stimuli with task-appropriate actions (Monsell 2003; Sakai 2008). Network models of task-switching typically represent context as a scalar input of unspecified biological origin (Dubreuil et al. 2022; Flesch et al. 2022; Pagan et al. 2025; Langdon and Engel 2025). Nevertheless, the hippocampus (HPC) has long been implicated in encoding context across many domains (Maren et al. 2013; Hirsh 1974; Smith and Mizumori 2006; Epstein et al. 2017; Cohen and Eichenbaum 1993; O’Keefe and Nadel 1978), positioning it as a plausible source of contextual signals that specify task sets during flexible behavior.

Activity-dependent tagging provides a powerful approach to test the causal role of context-dependent HPC representations. It enables selective labeling and manipulation of HPC neurons active during contextual learning (referred to as “engrams”) (Josselyn and Tonegawa 2020). Foundational studies demonstrated that artificially reactivating HPC engrams can drive context-dependent behaviors (Garner et al. 2012; Liu et al. 2012; Ramirez et al. 2013; Coelho et al. 2024; Redondo et al. 2014). Yet to our knowledge, these demonstrations have all been performed within a single task set. For example, in a typical context-dependent place preference engram study the rule linking sensory stimulus and action is fixed throughout the experiment (“sensory stimulus indicating context A → go to place X”). A similar description applies to fear conditioning and place avoidance engram studies. Whether or not HPC engrams can causally drive task sets thus remains unresolved, with important implications for both the engram and cognitive control literatures.

Moreover, HPC neurons are not themselves motoneurons. To affect behavior, HPC engram reactivations must influence either downstream motor representations themselves, or the computations that lead to motor activity. How this occurs, and the specific pathways involved, also remains unknown. Activity-dependent fluorescent labeling across the brain during HPC engram reactivation has implicated a broad spectrum of brain regions as potential downstream targets of the reactivation (Roy et al. 2022; Dorst et al. 2024; Tanaka et al. 2014; Guskjolen et al. 2018). But to our knowledge, no study has yet simultaneously reactivated HPC engrams and recorded downstream cortical activity during behavior, leaving the downstream neural dynamics by which engrams drive context-dependent behavior largely uncharacterized. This gap is especially critical because engram reactivation studies typically impose artificially synchronous activity (e.g., optogenetically), potentially obscuring how engrams operate under natural conditions and how their activity is translated into behaviorally-relevant downstream signals.

Existing data thus leave several critical questions entirely open (Fig. 1A). First, are HPC engram reactivations restricted to driving context-dependent motor actions? Or are they capable of driving context-dependent task sets, which are fundamentally more abstract? Second, do behaviorally-relevant downstream effects occur on a timescale of seconds, consistent with synaptic activations? Or over minutes, consistent with slower neuromodulatory processes? Third, can they drive explicit representations of context in downstream regions? Or do they influence downstream computations without modifying existing context representations? And fourth, does the artificially synchronous HPC stimulation used in most engram reactivation studies lead to downstream activity that preserves core features of natural spatiotemporal circuit dynamics? Or are the downstream natural dynamics fundamentally disrupted?

Here we set out to answer these questions with a combination of a novel mouse task-switching behavior, HPC engram reactivation, and simultaneous electrophysiological recordings in one potential downstream target, the medial prefrontal cortex (mPFC) (Thierry et al. 2000; Hoover and Vertes 2007; Ye et al. 2017; Eichenbaum 2017). We chose the mPFC as a strong candidate for observing downstream effects of HPC engram reactivation because decades of research have established that the PFC is required for task set updating (Durstewitz et al. 2010; Birrell and Brown 2000; Milner 1963; Miller and Cohen 2001), and coordinated HPC–mPFC activity is critical for memory-guided planning and decision-making more broadly (Wikenheiser et al. 2017; Weilbächer and Gluth 2016; Tavares and Tort 2022; Tang et al. 2021; Elston and Wallis 2025; Jadhav et al. 2016; Eichenbaum 2017; Spellman et al. 2015; Place et al. 2016).

Our results show that HPC engrams are capable of driving downstream task sets, without directly driving motor representations; they do so within hundreds of milliseconds of reactivation, thus establishing a key role for synaptic and network effects in the engram→behavior pathway; they modify existing context representations; and despite the artificial nature of laser stimulation, they preserve major downstream spatiotemporal dynamics features, suggesting that the artificial aspects of the reactivation are filtered by the network into downstream activity that closely mimics natural activity.

## A mouse task-switching behavior

**Figure 1.**
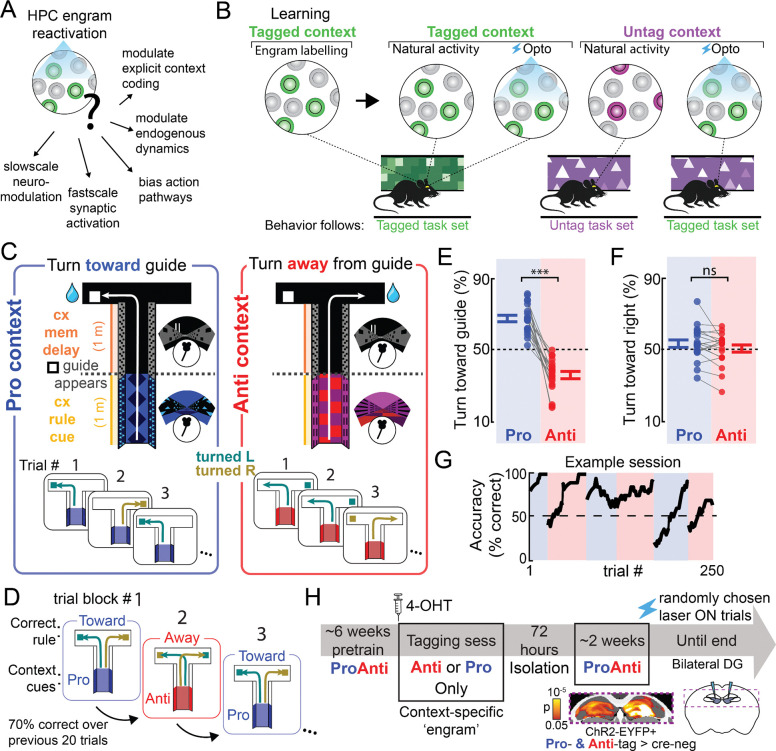
Pro-Anti task-switching paradigm and predictions. **(A)** Possible downstream mechanisms by which HPC engrams drive context-dependent behavior. **(B)** During context-dependent decision-making, the same HPC ensemble is predicted to be active during laser-off and opto trials in the tagged context, producing behavior that follows that the tagged task set. In the untagged context, different ensembles are predicted to be active during laser-off and opto trials, causing behavior to follow the tagged task set only during opto engram reactivation. **(C)** Mice performed a behavior involving different sensorimotor rules in different contexts: in the Pro context (blue), the rule was to turn toward the visual turn guide; in the Anti context (red), turn away from it. The context rule cue was presented only in the first half of the stem, followed by the context memory delay during which the guide appeared. In the delay the two contexts were identical to each other, requiring contextual identity to be held in memory from that point on. Guide position (left vs. right side of the T-maze) was balanced across trials. Cyan and gold refer to separate left and right choice trials, respectively. **(D)** Contexts were presented in interleaved blocks, and switched once performance exceeded 70% correct over the previous 20 trials. **(E)** Decision rule by context (probability of turning toward the guide) during pre-tagging sessions. Mice turned toward in Pro and away in Anti (N=20; ***p=2×10^−6^). **(F)** Motor choice (probability of right turn) was similar across contexts (p=0.26). **(G)** Example session showing performance (% correct, 10-trial within-block moving mean). **(H)** Experimental timeline. Bottom: significance map of ChR2–EYFP expression (Pro & Anti-tag > Cre- controls; N = 12 vs. 4) in HPC, overlaid on atlas (p < 0.05, SVC). In (E-F), dots represent individual mice; error bars denote mean ± SEM.

We first set out to answer whether HPC engrams can causally drive task sets (Fig. 1B). To dissociate motor action from task set specification, we designed a task-switching behavior in which context determines which sensorimotor decision rule, rather than motor action, to apply on each trial. Following task-switching paradigms in non-human primates (Munoz and Everling 2004; Mante et al. 2013; Wu et al. 2020; Elston and Wallis 2025; Siegel et al. 2015; Bernardi et al. 2020), and their adaptation to rodents (Pagan et al. 2025; Duan et al. 2015, 2021; Wimmer et al. 2015), we developed a behavioral task for head-fixed mice in virtual reality (VR) based on the classic “Pro/Anti task-switching” paradigm, in which context determines whether subjects orient toward or away from a stimulus (Fig. 1C).

Mice ran up the stem of a VR T-maze, and were trained to make a decision as to whether to turn left or right at the top of the “T”. The T-maze could be rendered in one of two contexts (labelled “Pro” and “Anti” in Fig. 1C), each of which was associated with a different sensorimotor rule. The contexts were distinguished by salient multisensory cues presented exclusively along the first half of the T-maze stem (the “rule cue” zone), and were presented in interleaved blocks of trials within each experimental session (Fig. 1D). Upon exiting the rule cue zone and entering the second half of the stem (referred to as the “contextual memory delay” zone), a salient visual stimulus called a turn “guide” was presented either on the left end of the VR corridor or on the right end (balanced across trials). The guide was visible throughout the memory delay zone of the T-maze stem. In the Pro context, the task-appropriate sensorimotor rule for receiving water reward was to turn *toward* the guide at the top of the stem (regardless of whether the guide was on the left side or right side). In the Anti context, the task-appropriate sensorimotor rule was to turn *away* from the guide. The memory of the context thus did not specify a particular “go-left” or “go-right” motor action, but instead specified the rule to apply.

Once trained, mice showed a strong effect of context on their decisions (Fig. 1E; for training procedure, see Fig. S1). Consistent with the task design, there was a negligible effect of context on their overall rightwards versus leftwards choices (Fig. 1F). Within each session, mice ran a median (± 1MAD) of 280±37 trials, and completed 4±1 context blocks (Fig. 1G; Fig. S2A). Control manipulations of the contextual cues confirmed that these multisensory cues played a role in how mice identified context and retrieved the associated decision rule, rather than exclusively inferring the current decision rule from recent trial outcome feedback (Fig. S2E–G). Switching contexts across blocks incurred a transient performance cost, consistent with perseveration of the previous context’s decision rule (Fig. 1G; Fig. S2B). Asymmetric task switch costs are often found in other species (Monsell et al. 2000; Allport et al. 1994; Duan et al. 2015); here the switch cost was greater when switching from Pro to Anti than vice versa (Fig. S2C–D).

## HPC engrams drive context-dependent sensorimotor task sets

To test whether context engrams contribute to this task-switching behavior, we used activity-dependent optogenetics to label and later reactivate HPC ensembles associated with either the Pro or the Anti context. We crossed mice expressing tamoxifen-dependent CreERT2 under the *Arc* promoter with a Cre-dependent ChR2-EYFP reporter line, so that neurons active shortly following tamoxifen injection permanently express ChR2 (Denny et al. 2014; Guenthner et al. 2013). To target context-specific ensembles and avoid environmental novelty-related ensembles (Cleland et al. 2017), mice were first pretrained on the ProAnti task (Fig. S1A). They were then injected with 4-hydroxytamoxifen (4-OHT) and completed one training session that consisted of trials of only a single context (Fig. 1H). In the “Pro-tag” group of mice, this session had only Pro context trials; In the “Anti-tag” group, this session had only Anti context trials. This single-context training session labeled neurons active in their specific tagging context with ChR2-EYFP (Fig. S3A).

Behavioral performance improved during the tagging session (Fig. S1B), confirming that animals continued to strengthen their association between a context and its corresponding decision rule. Following a three-day isolation period, mice were retrained to criterion. In subsequent sessions, the tagged population was reactivated during select task epochs by delivering blue laser light (~2 mW, 20 Hz) through bilateral optic fibers targeting the dorsal dentate gyrus (DG; Fig. S1C, S3B). This was done on 30% of randomly selected trials, with the remaining interleaved 70% of trials serving as laser-off controls. To describe both Pro-tag and Anti-tag groups of mice together, we will refer to the context matching an animal’s tagging context as the “tagged” context, and the other context as the “untagged” context.

**Figure 2.**
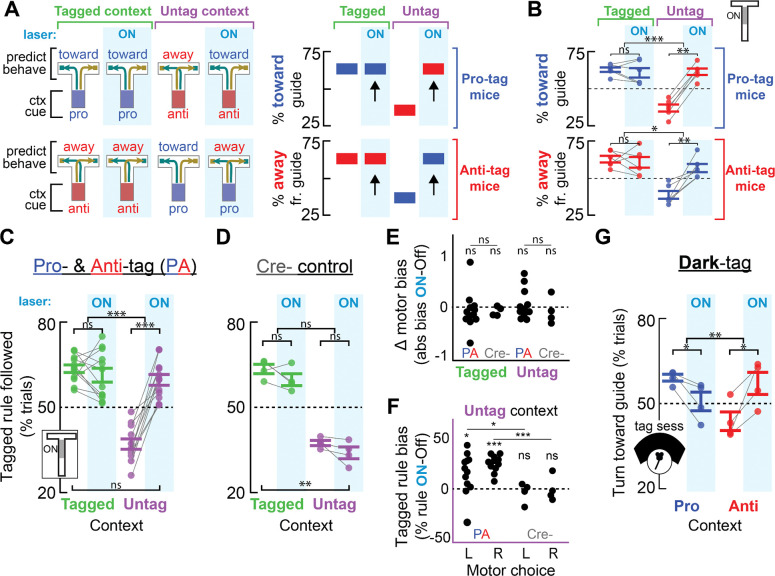
Context-specific hippocampal engrams bias context-dependent decisions. **(A)** Predicted behavioral effect: reactivating tagged engrams should bias mice to use the tagged choice rule in both Tagged and Untagged contexts, in both Pro- (top row) and Anti- (bottom row) tagged mice. Behavior is thus predicted to be tag-consistent (black arrows) independent of the experimenter cued context. **(B)** Observed: memory delay laser stimulation increased use of the tagged rule in the Untagged context, but there was no effect of the laser in the Tagged context, in both Pro-tag (top, N=6) and Anti-tag (bottom, N=6) mice. **(C)** Combining Pro- and Anti-tag mice (PA, N=12), memory delay stimulation increased use of the tagged rule in the untagged context (above Off, p=0.000001, and above 50% chance, p=0.0004). In the tagged context, PA used the tagged rule during both laser-off and -on trials, with no additional effect of the laser (context × laser interaction: p=0.00002). **(D)** Cre- controls (N=4) showed no laser effects in either context. **(E)** No changes in absolute motor-choice bias within or across groups in either context. **(F)** Tagged rule bias in the Untag context in PA present during both left and right choice trials (both p<0.02), absent in Cre-. **(G)** Dark-tag controls (N=4), tagged in darkness without a task, were generally impaired by laser stimulation (context × laser interaction: p=0.007). Dots denote individual mice; error bars denote mean ± SEM. *p<0.05, **p<0.01, ***p<0.001. Full statistics in Table S1.

If HPC engrams contribute to flexible decision-making, reactivating context-specific ensembles should retrieve the associated decision rule and bias choices accordingly. During tagged context trials, behavior during laser-on trials could be similar to behavior during laser-off (control) trials, particularly if HPC activity was already correctly representing the tagged context before the laser is turned on. In contrast, during untagged context trials, turning the laser-on should cause mice to flip from the untagged to the tagged representation, and disproportionately apply the tagged rule. We thus expect the most salient effects to be seen in the untagged context. Overall, the pattern of predicted effects is depicted in Fig. 2A.

Fig. 2B–C shows the experimentally obtained effects when context-specific HPC ensembles were optically stimulated during the contextual memory delay (i.e., the period during which mice must maintain context in memory to guide their choice; effects of rule cue zone reactivation are described below). The observed effects were remarkably similar to the predictions (compare Figs. 2A and 2B). Strikingly, choices on laser-on trials in the untagged context were indistinguishable from choices on laser-off trials in the tagged context, indicating that artificial reactivation substituted for the natural contextual cues. We found no significant effect of reactivation in the tagged context, suggesting that the strength of an already-represented tagged context was not boosted further by optical reactivation. In sum, reactivation of HPC engrams in the memory delay zone drove tag-consistent flexible decision-making: turning the laser-on led mice to behave as if they were in the tagged context, even when tested in the opposite context.

To confirm that the tag-specific decision bias was not an artifact of optical stimulation alone, we repeated the experiment in control mice that underwent the same procedures but lacked the Cre allele (Cre- group). In these Cre- mice, optical stimulation had no effect (Fig. 2D), ruling out nonspecific stimulation effects and demonstrating that the behavioral bias depended on tagged HPC ensembles.

The context-specific bias induced by HPC memory delay zone reactivation was independent of which context was tagged, with similar effects in both the Pro- and Anti-tag groups (Fig. 2B; Fig. S4A–B). The tag-specific decision bias was robust across sessions (observed in 87.8% of sessions; sign test, p=1.37×10^−8^; Fig. S4C–D) and across trials within a block, with reactivation causing tag-consistent choices to occur more frequently in the untagged context regardless of time since context switching (Fig. S4E). Control analyses confirmed that this effect was not due to nonspecific changes in VR locomotion (Fig. S5).

To test whether reactivation of context-specific HPC cells drives decision rule retrieval rather than simply biasing motor output, we first confirmed that optical stimulation did not alter overall motor choice bias (Fig. 2E). We then calculated the decision rule bias separately for left- and right-turn choice trials. Reactivation of context-specific HPC ensembles caused mice to apply the tagged rule in the untagged context for both turn directions (Fig. 2F). These results show that HPC engram reactivation influences retrieval of the decision rule itself, rather than producing a motor bias.

If HPC engrams provide a neural source of context during flexible decision-making, activating HPC cells unrelated to a task-relevant context should disrupt performance. To test this, we applied the same experimental procedure to a separate group of mice, except that following 4-OHT treatment to induce tagging, these mice were head-fixed in the VR rig in the dark and received randomly interspersed rewards (Dark-tag group; Fig. S6A–D). For this Dark-tag group, memory delay zone reactivation produced choices that were at chance in both contexts (Fig. 2G), a markedly different effect compared to the Pro- or Anti-tagged mice. Overall performance (% correct) during laser-off trials was also reduced in Dark-tag mice relative to pre-perturbation sessions (Fig. S6E). In a separate group of VGAT-ChR2 mice, optogenetic inhibition broadly targeting the DG also generally impaired ProAnti behavior (Fig. S6F). These results indicate that the context-specific decision bias in Pro- and Anti-tag mice requires activation of a HPC ensemble associated with the tagging context, and that stimulating a HPC population unrelated to decision-making interferes with general task performance rather than eliciting context-specific decisions.

The preceding results involved reactivation of context-specific HPC cells during the memory delay zone of the stem of the T-maze, a period during which mice must maintain contextual information in memory to guide their choices. To test whether HPC engrams also contribute during other task epochs, we also reactivated HPC engrams in Pro- and Anti-tag mice during the whole stem of the VR T-maze (i.e., both the rule cue and memory delay zones), or during the rule cue zone alone. Whole-stem reactivation biased choices toward the tagged rule similar to memory delay reactivation alone (Fig. S7A). But in contrast, reactivation during the rule cue zone failed to produce a tag-specific bias: behavior on laser-on trials was impaired in both contexts, with the strongest deficit in the Anti context regardless of tag identity (Fig. S7B–C). Rule cue-only reactivation also decreased mouse running speed specifically at the transition between the rule cue zone and the memory delay (Fig. S5B). Memory delay stimulation produced a significantly stronger tag-specific bias than rule cue–only reactivation (p=0.0002; Fig. S7D), indicating that HPC engram activity is particularly critical for decision-making when contextual information must be internally maintained and a context-dependent choice is made.

## HPC engrams drive mPFC contextual representations

**Figure 3.**
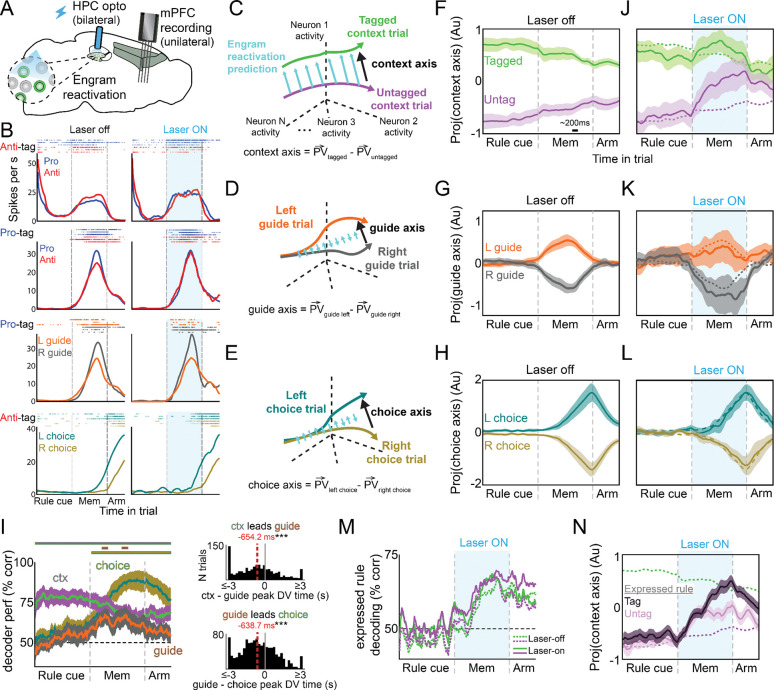
HPC engram reactivation reinstates tagged context representation in mPFC. **(A)** mPFC single-unit activity recorded using Neuropixel probes during simultaneous ProAnti behavior and HPC engram reactivation (N=2 Pro-tag, N=2 Anti-tag). **(B)** Example mPFC single-unit activity during laser-off (left) and memory-delay reactivation (right) trials for four different units (rasters from 6 random trials). **(C-E)** Schematics of context, turn guide side, and choice coding axes, and predicted effects of engram reactivation. (**F-H**) Population trajectories over time projected onto the (F) context axis, (G) guide axis, (H) and choice axis during correct laser-off trials (N=11 sessions). Scale bar in (F) indicates elapsed time during the memory delay. **(I)** Left: Trialwise LDA decoding of context, turn guide side, and choice over time during laser-off trials. Significance markers show above-chance (50%) decoding (p<0.001). Right: on individual trials, the time of the absolute peak of the context decision-variable (DV) preceded guide DV (top: sign-test, p=3.25×10^−30^) and guide preceded choice DV (bottom: p=5.86×10^−26^). (**J-L**) Projections onto the (J) context, (K) guide, (L) and choice axes during memory delay reactivation trials. Dotted lines denote respective laser-off baselines. **(M)** LDA decoding of expressed decision rule (Pro vs. Anti) during laser-off (dotted) and memory delay reactivation (laser-on; solid) trials, shown separately by context. Decoders were trained on laser-off trials controlling for contextual cues, motor choice, and performance. No significant differences between laser-on and -off trials in either context, indicating that the laser induced shift in expressed rule was represented in mPFC. Curves show bootstrap mean; overlapping error bars omitted for clarity. **(N)** Same as (J) in the untagged context, but split by expressed behavioral rule (mean ± SEM pooled across trials; statistical inference via bootstrap). (F-L) shaded lines denote bootstrap mean ± SD. Full statistics in Figs. S10–S12, and Table S1.

Building on these behavioral findings, we next sought to determine how HPC engram reactivation shapes downstream neural computations that implement task sets. We chronically recorded single-unit activity in the mPFC during simultaneous ProAnti task performance and HPC engram reactivation (Fig. 3A), allowing us to directly examine how context-specific HPC output influences prefrontal representations supporting flexible behavior. Recordings targeted anterior cingulate and prelimbic cortices (296 ± 82 simultaneously recorded units, median ± s.d., per behavioral session; Fig. S3C).

**Figure 4.**
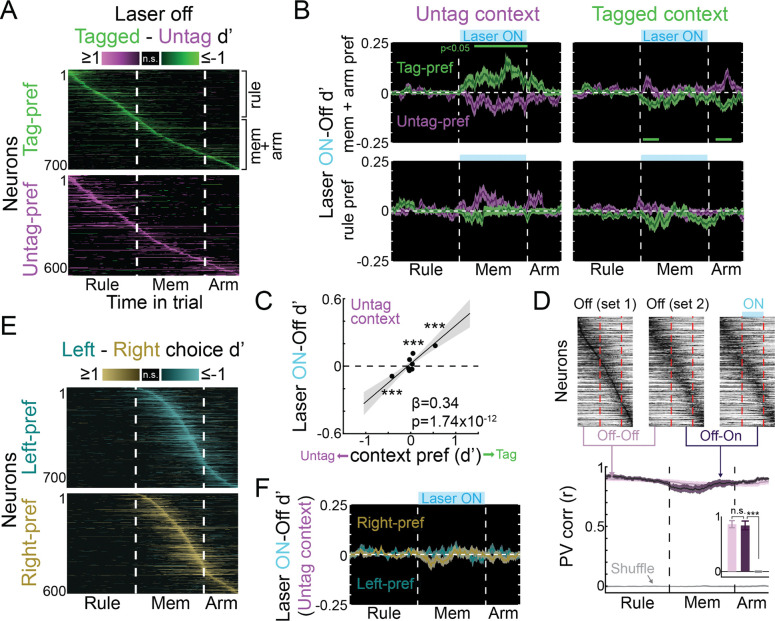
Single-unit contributions to HPC-driven context reinstatement in mPFC. **(A)** Context discriminability (d′) in context modulated cells during laser-off trials (inclusion threshold: p<0.01, uncorrected). Neurons are sorted by peak discriminability time and context preference. **(B)** Laser modulation (on–off d′) for neurons grouped by rule cue or memory and arm field location, and context preference, as in (A), shown separately in untag and tagged contexts (median ± bootstrap SEM). During engram stimulation in the untagged context, mem + arm tag-preferring neurons were excited (p<0.05, FWE-corrected), whereas mem + arm untag-preferring neurons showed weaker, non-significant suppression. Reactivation in the tagged context produced brief suppression of tag-preferring neurons at laser onset and offset. Rule cue neurons were not significantly modulated. **(C)** Mean laser modulation vs. laser-off context discriminability during the 2nd half of the memory delay, pooled across neurons (untag context; n=1992). Laser effects scaled with context tuning: untag-preferring neurons were significantly suppressed and tag-preferring neurons excited (linear mixed model, shading denotes 95% CI). Points show means of eight equally sized context preference bins (sign-rank, FDR-corrected: bin #1 p=4.9×10^−5^, 2–6: >0.05, 7: 0.0007, 8: 1.1×10^−8^). **(D)** Top: Untag context activity traces sorted by peak firing time during an example laser-on–trial-matched subset of laser-off trials (set 1). Rows are max-normalized per condition. Bottom: time-resolved population vector (PV) correlation across conditions (mean ± bootstrap SEM). Shuffle permutes neuron identities in laser-off activity for the laser-off vs. -on comparison. Inset shows average similarity over the memory delay (±1 SD; t-tests: off vs. on: p=0.35, on vs. shuffle: 9.9×10^−12^). **(E)** Same as (A) split by choice preference. **(F)** No laser modulation in choice-preferring neurons during stimulation in the untag context. *p<0.05, **p<0.01, ***p<0.001.

Consistent with previous studies involving rodent navigation (Kaefer et al. 2020; Hok et al. 2005; Zielinski et al. 2019; Sauer et al. 2022), we found that mPFC had substantial place-field-like activity, with most neurons having a preferred location of space in which they tended to have high activity. As the animal traverses the maze, this led to an orderly sequence of activated neurons that tiled the T-maze (example neurons Fig. 3B and Fig. S8A; see Fig. 4 for further characterization of these sequential dynamics). Successful performance of the ProAnti task requires animals to integrate contextual information with turn-guide side to select the appropriate motor response on each trial. Consistent with nonhuman primate and rat studies (Rigotti et al. 2013; Mante et al. 2013; Euston et al. 2012; Spellman et al. 2015; Duan et al. 2021), during laser-off trials, individual mouse mPFC neurons encoded these task variables, including context, turn-guide side, and upcoming choice. The variables were encoded as modulation of the underlying place-dependent activity (Fig. 3B; S8A–B).

To characterize how these key task variables were represented at the population level, we analyzed laser-off activity in neural state space, with each dimension corresponding to a single neuron. We identified the coding directions in this space that maximally discriminated context, turn guide side, and upcoming choice (Inagaki et al. 2019; Li et al. 2016) (Fig. 3C–E). These coding directions were largely orthogonal to each other, indicating that each of these task variables was represented along distinct axes of population activity (Fig. S9).

Projecting single-trial population activity onto the context axis revealed well-separated context-specific distributions throughout the entirety of laser-off trials (Fig. 3F; S10A). In contrast, projections onto the guide and choice axes diverged following the appearance of the turn guide (Figs. 3G–H; S11A; S12A). Linear discriminant analysis (LDA) revealed a temporal ordering to the emergence of these representations on individual trials: consistent with subjects using the Pro/Anti trial block structure, information about context was present from the start of each trial; upon the start of the memory period/presentation of the turn-guide, a representation of turn-guide side appeared, followed by a representation of motor choice (Fig. 3I). Thus, mPFC population activity mirrors the sequence of computational task demands, representing context, turn guide, and motor choice in the order expected for accurate performance. We also asked whether mPFC activity encoded the decision rule that the animals expressed in their behavior. Because the animals made errors, we defined the expressed decision rule on each trial as Pro if the animal turned towards the guide, and as Anti if the animal turned away from it, independent of the rule cued by the experimenters. LDA found reliable decoding of the expressed decision rule in laser-off trials, even when controlling for contextual cues, motor choice, and balancing the number of correct and incorrect trials used in the analysis (Fig 3M). Thus, during control trials, both the cued decision rule and the expressed decision rule are represented in mPFC during the memory delay, with the latter ramping up during the memory delay.

Having established that mPFC encodes the variables required to solve the ProAnti task, we next asked whether and how these representations are causally influenced by HPC engram reactivation. If HPC engrams bias motor action pathways, we would expect perturbations along the choice axis. Alternatively, if HPC reactivation conveys contextual information that specifies the active task set, its primary effect should be selective to the context axis. In this latter framework, HPC reactivation would influence behavior by altering the contextual state from which choice computations proceed, rather than by directly modifying motor choice representations themselves. Since choice representations emerged following context, activity along the choice axis itself may thus remain largely unperturbed despite reliable changes in choice behavior.

mPFC neurons were not directly activated by HPC laser stimulation (Fig. S13), indicating that any observed effects reflected network-level modulation, likely through polysynaptic pathways (Eichenbaum 2017). During memory-delay stimulation, HPC engrams selectively altered activity along the context axis: In the untagged context (purple traces in Fig. 3I), reactivation shifted mPFC population activity along the context axis toward the tagged-context trajectory, whereas no significant effect of the laser was observed in the tagged context (green in Fig. 3I). By the end of the memory delay in laser-on trials, tagged and untag context trajectories became statistically indistinguishable (Fig. S10A). Across trials, HPC-driven contextual reinstatement in mPFC occurred at variable times throughout the memory delay, with a median shift time of ~850 ms following laser onset (Fig. S14A). Importantly, the context axis accounted for a comparable fraction of population variance during laser-off and laser-on trials (Fig. S10B), demonstrating that engram reactivation did not change the contribution of context coding but instead biased activity within this subspace. Thus, memory delay reactivation reinstated an explicit engram-associated context representation in mPFC.

In contrast, trajectories projected onto the guide and choice axes remained well separated during memory-delay HPC reactivation, with no significant effect of laser stimulation on how these variables are represented, either overall (Fig. 3J–K; S11A; S12A) or when analyzed separately in each context (Fig. S11B–C; S12B–C). In other words, activity along the choice axis aligned with the animal’s expressed choice in the untagged context, even when HPC reactivation caused a change in expressed rule (Fig. S12D). Notably, on trials where HPC reactivation caused mPFC contextual reinstatement, robust choice representations emerged following that reinstatement (Fig. S14B), suggesting that laser effects on choice are mediated specifically through effects on the represented context. Guide representations were unrelated to the timing of contextual reinstatement (Fig. S14C). These results are consistent with the idea that an HPC-driven contextual state in mPFC gates the emergence of downstream choice representations.

Consistent with the nonspecific behavioral effects of rule-cue-period-only stimulation, ambiguous neural effects were observed in mPFC context representations for trials with engram reactivation during this epoch. This was particularly salient in the Anti context regardless of tag identity (Fig. S15A–D). As during memory-delay-period HPC stimulation, rule cue reactivation had no significant effect on the guide and choice dimensions (Fig. S15E–F).

If HPC engrams establish the contextual input for flexible decision-making, then reinstating context in mPFC should place the network into a state consistent with its behavior on that trial. To test this, we asked whether the expressed rule decoder, trained on laser-off trials, also decoded the expressed rule during laser-on trials. We found that this decoding was intact: as with guide and choice, HPC engram reactivation had no significant effect on how the expressed rule was represented (Fig 3M). To ask what distinguished trials in which reactivation did or did not induce a change in expressed rule, for each of these two groups of trials we examined the time-course of the context representation during laser-on trials in which the cued context was the untagged context. We found that trajectories along the context axis initially shifted toward the tagged context representation regardless of the expressed rule. Yet, this shift only persisted on trials in which the tagged rule was ultimately expressed (i.e., when cued and expressed rules did not match, Fig. 3N; Fig. S14D). When reactivation failed to induce the tagged decision rule (i.e., when cued and expressed rules matched), population activity returned to the untagged trajectory by the end of the trial. These results indicate that HPC engrams transiently reinstate the tagged context in mPFC independently of behavior, but that only sufficiently sustained contextual bias leads the network to stabilize in a tag-rule-consistent state.

These data demonstrate that HPC engrams modulate mPFC computations on a timescale of hundreds of milliseconds. To provide an independent, mechanistically-direct readout of the timing of HPC→mPFC transmission, we also examined the entrainment of mPFC spiking to the 20-Hz HPC stimulation frequency. Fourier analysis of single-neuron spike trains revealed a robust 20-Hz peak during laser-on relative to laser-off trials, demonstrating frequency-specific entrainment of mPFC to HPC-driven activity (Fig. S16A–C). Time-resolved wavelet analysis further showed that engram reactivation elicited a rapid, broadband increase in mPFC spiking ~100 ms after laser onset, likely reflecting a transient increase in mPFC excitability, followed by disproportionate entrainment of mPFC spiking to the 20-Hz stimulation frequency emerging ~800 ms after laser onset (Fig. S16D–F). Together, the contextual representation and entrainment results converge on the conclusion that HPC engrams drive mPFC contextual reinstatement through multisynaptic transmission and rapid (hundreds of milliseconds) network effects.

The HPC-driven population-level contextual reinstatement observed in mPFC could, in principle, arise through excitation of neurons preferring the tagged context, suppression of neurons that prefer the untagged context, or a combination of both. It could also reflect changes in a small set of highly responsive neurons or distributed modulation across a broader context-tuned ensemble. To distinguish these possibilities, we examined the contribution of context to single-neuron mPFC activity (Fig. 4A). During memory-delay reactivation in the untagged context, neurons preferring the tagged context and with a firing field in the memory delay zone or arm of the T-maze exhibited a robust increase in firing rates (Fig. 4B, left upper). Conversely, neurons preferring the untagged context showed firing rate suppression, albeit with weaker modulation than tagged-preferring cells. No effect of memory delay zone reactivation was seen in cells with firing fields in the earlier rule zone (Fig. 4B, left lower). As a result, population activity during stimulation shifted toward a pattern resembling that observed in the tagged context during laser-off trials. This shift reflected a coordinated, population-wide modulation that scaled with each neuron’s degree of context tuning, rather than being driven by a small subset of highly responsive units (Fig. 4C).

HPC reactivation in the tagged context produced only brief, weak suppression of tag-context-preferring neurons following laser onset and offset without sustained population-wide modulation (Fig. 4B, right). This is consistent with the absence of HPC stimulation effects on mPFC population activity during tagged context trials (Fig. 3J). Neuronal activity was also not modulated as a function of their choice tuning (Fig. 4E, F). Finally, reactivation in the rule cue zone failed to elicit tag-consistent modulation, instead biasing activity toward a more Pro-context-like pattern in the Anti context regardless of tag identity, paralleling the observed change in behavior (Fig. S15G).

The lack of an effect of memory-delay HPC reactivation on mPFC neurons with firing fields in the rule cue zone suggests that reactivation may bias mPFC contextual representations without reorganizing spatiotemporal sequence dynamics. Indeed, direct examination of mPFC sequential activity revealed that although a small number of neurons altered their sequential firing during reactivation (Fig. S8C), spatiotemporal sequences were strongly preserved at the population level (Fig. 4D). Bayesian decoding of spatiotemporal position from mPFC activity confirmed this observation: decoders trained on laser-off trials transferred to reactivation trials with comparable accuracy, with only a small increase in decoding error comparable to the error observed when decoding position across contexts during laser-off trials (Fig. S17A–B). This error increase during HPC stimulation was driven by a small (~3.4 cm) systematic backward shift in decoded position, consistent with a small spatiotemporal advance in sequential firing rather than disruption of the sequence itself (Fig. S17C). Rule cue reactivation had no significant effect on sequential activity (Fig. S17D–F). Thus, despite the artificial HPC stimulation lasting the entirety of the memory delay, and the observed 20-Hz entrainment, engram reactivation modulated the contextual component of mPFC activity without altering sequential dynamics. This suggests that the artificial aspects of the reactivation are filtered by the network into downstream activity that closely resembles natural activity.

## Discussion

In this study, we show that HPC engrams can causally drive behaviors that require abstract, flexible task sets. Rather than eliciting specific motor action plans, reactivation of context-specific HPC engrams led mice to retrieve and apply the appropriate rule for a decision, even when that rule demanded opposing actions across trials. Therefore, engram reactivation can direct the subject’s mapping between sensory inputs and actions, the defining feature of a task set. By demonstrating that HPC engrams are causal to flexible behaviors guided by abstract cognitive states, the results presented here extend prior work that showed that reactivation of sensory- or fear-associated engrams are sufficient to direct motor actions (Liu et al. 2012; Ramirez et al. 2013; Coelho et al. 2024; Redondo et al. 2014).

Previous studies combining HPC engram stimulation with immediate early gene (IEG) mapping in downstream regions have shown that HPC engram reactivation propagates activity through HPC circuits and engages distributed downstream targets (Roy et al. 2022; Dorst et al. 2024; Tanaka et al. 2014; Guskjolen et al. 2018). However, how that reactivation-driven activity relates to behavior remains unclear. With regard to mPFC, for example, (Roy et al. 2022) found that mPFC was one of the regions showing decreased neural activity due to HPC engram inhibition. But whether or not that engram-modulated activity relates to behavior or task representations, and if so, how, cannot be determined from IEG mapping alone. Only a small number of reports have used HPC engram reactivation while recording downstream neural activity during behavior (Coelho et al. 2024; Suthard et al. 2024; Rahsepar et al. 2023; Norman et al. 2025), and these focused purely within the HPC loop, for example reactivating engrams in one HPC subregion while recording in another HPC subregion. As a result, the fundamental question of how engram-driven activity shapes neocortical dynamics and population codes to support ongoing behavior has remained unexplored. Here, we show that engram reactivation selectively shifts mPFC population activity along a task-relevant axis predictive of flexible decision-making, thus linking circuit-level reactivation to cognitive function.

HPC reactivation could influence behavior either by biasing downstream motor representations directly or by reinstating an abstract contextual state that specifies the active task set. In the former case, effects should appear along motor-choice-related neural dimensions, whereas in the latter, they should be confined to context-related dimensions, with changes in choice emerging from the altered contextual state. Our results are consistent with the latter account: HPC engram reactivation selectively shifted mPFC activity toward the tagged context representation observed during control trials, effectively reinstating a tag-like contextual state. This finding demonstrates that reactivation of context-specific HPC engrams is sufficient to drive mPFC representations of context, suggesting a candidate mechanism for the context-dependent remapping previously observed in mPFC (Jung et al. 1998; Sauer et al. 2022).

In contrast, choice representations in mPFC remained essentially intact following HPC engram reactivation, in the sense that they remained aligned with the animal’s expressed choice even when HPC reactivation caused a change in expressed rule. mPFC sequential activity dynamics and guide representations were also preserved. Thus, despite the highly artificial nature of the HPC laser stimulation (15 ms pulses at 20 Hz throughout the memory zone) and despite the observed 20 Hz entrainment in mPFC activity, neural dynamics in mPFC were remarkably similar to their natural dynamics in control trials. The main effect of HPC laser stimulation was restricted to a change in context representation. Even along the mPFC context coding axis itself, HPC laser stimulation did not cause activity outside the control range, and activity variance explained by this axis remained the same on laser-on versus laser-off trials. These results suggest that the system is robust to artificial stimulation, filtering it into dynamics closely aligned with its own endogenous dynamics.

Several anatomical pathways could mediate HPC’s influence over mPFC, including polysynaptic routes through thalamic, entorhinal, and retrosplenial circuits (Eichenbaum 2017). Identifying the mechanisms by which engrams modulate downstream activity to drive behavior will be greatly facilitated by identifying the timescale of the effects. Nevertheless, most prior experiments consist of minute-scale stimulation epochs (Liu et al. 2012; Ramirez et al. 2013; Coelho et al. 2024; Redondo et al. 2014), which can limit temporal resolution when assessing how engram activity impacts downstream circuits. Here, the rapid emergence of mPFC entrainment and contextual reinstatement following HPC stimulation indicates that engram-driven effects can arise through network interactions on the timescale of hundreds of milliseconds.

Notably, engram reactivation induced a context-specific rule bias only when stimulation occurred during the contextual memory delay, not during the rule cue period when contextual information was externally available. This dissociation suggests that HPC engrams may be particularly important for maintaining or reinstating task context during internally-guided decision-making, rather than during sensory identification of context. More generally, these findings suggest that the influence of HPC engrams on cortical task representations depends on behavioral state and task demands. Identifying the circuit pathways and network conditions that gate HPC-driven task set specification will be an important direction for future work.

More broadly, these results align with long-standing indexing theories of HPC function, which propose that HPC activity patterns serve as pointers capable of reinstating distributed cortical representations (Guskjolen and Cembrowski 2023; Teyler and DiScenna 1986). In this view, HPC engrams provide the contextual input that selects among competing rule representations, enabling rapid adjustment of behavioral policies. Selective modulation of downstream representational subspaces by HPC engrams may therefore reflect a general mechanism by which memory systems gate task set configuration, with implications for decisions ranging from approach–avoidance to inference and generalization.

## Supplementary Material

Supplement 1

## Data Availability

Upon publication, the datasets generated during the current study will be deposited in a public repository, and associated code will be made freely available.

## References

[R1] Allport D. Alan, Styles Elizabeth A., and Hsieh Shulan. 1994. “Shifting Intentional Set: Exploring the Dynamic Control of Tasks.” Attention and Performance 15 (945): 421–452.

[R2] Bernardi Silvia, Benna Marcus K., Rigotti Mattia, Munuera Jérôme, Fusi Stefano, and Daniel Salzman C.. 2020. “The Geometry of Abstraction in the Hippocampus and Prefrontal Cortex.” Cell 183 (4): 954–967.e21. 33058757 10.1016/j.cell.2020.09.031 PMC8451959

[R3] Birrell J. M., and Brown V. J.. 2000. “Medial Frontal Cortex Mediates Perceptual Attentional Set Shifting in the Rat.” The Journal of Neuroscience: The Official Journal of the Society for Neuroscience 20 (11): 4320–4324. 10818167 10.1523/JNEUROSCI.20-11-04320.2000 PMC6772641

[R4] Chiaruttini Nicolas, Castoldi Carlo, Requie Linda Maria, 2025. “ABBA+BraiAn, an Integrated Suite for Whole-Brain Mapping, Reveals Brain-Wide Differences in Immediate-Early Genes Induction upon Learning.” Cell Reports 44 (7): 115876. 40553651 10.1016/j.celrep.2025.115876

[R5] Cho Jounhong Ryan, Bolkan Scott S., Brown Lindsey S., 2025. “Striatal Pathways Oppositely Shift Cortical Activity along the Decision Axis.” In bioRxivorg. July 30. 10.1101/2025.07.29.667406.

[R6] Claudi Federico, Tyson Adam L., Petrucco Luigi, Margrie Troy W., Portugues Ruben, and Branco Tiago. 2021. “Visualizing Anatomically Registered Data with Brainrender.” eLife 10 (March). 10.7554/eLife.65751. PMC8079143 33739286

[R7] Cleland J. P., Willis E. F., Bartlett P. F., and Vukovic J.. 2017. “Somatic Arc Protein Expression in Hippocampal Granule Cells Is Increased in Response to Environmental Change but Independent of Task-Specific Learning.” Scientific Reports 7 (1): 12477. 28963515 10.1038/s41598-017-12583-1 PMC5622137

[R8] Coelho Cesar A. O., Mocle Andrew J., Jacob Alex D., 2024. “Dentate Gyrus Ensembles Gate Context-Dependent Neural States and Memory Retrieval.” Science Advances 10 (31): eadn9815. 39093976 10.1126/sciadv.adn9815 PMC11296340

[R9] Cohen Neal J., and Eichenbaum Howard. 1993. Memory, Amnesia and the Hippocampal System. MIT Press.

[R10] Cokelaer T., Haschet Juergen. 2017. “'Spectrum': Spectral Analysis in Python.” Journal of Open Source Software 2(18), 348

[R11] Denny Christine A., Kheirbek Mazen A., Alba Eva L., 2014. “Hippocampal Memory Traces Are Differentially Modulated by Experience, Time, and Adult Neurogenesis.” Neuron 83 (1): 189–201. 24991962 10.1016/j.neuron.2014.05.018 PMC4169172

[R12] Dorst Kaitlyn E., Senne Ryan A., Diep Anh H., 2024. “Hippocampal Engrams Generate Variable Behavioral Responses and Brain-Wide Network States.” The Journal of Neuroscience: The Official Journal of the Society for Neuroscience 44 (2). 10.1523/JNEUROSCI.0340-23.2023. PMC10860633 38050098

[R13] Duan Chunyu A., Erlich Jeffrey C., and Brody Carlos D.. 2015. “Requirement of Prefrontal and Midbrain Regions for Rapid Executive Control of Behavior in the Rat.” Neuron 86 (6): 1491–1503. 26087166 10.1016/j.neuron.2015.05.042

[R14] Duan Chunyu A., Pagan Marino, Piet Alex T., 2021. “Collicular Circuits for Flexible Sensorimotor Routing.” Nature Neuroscience 24 (8): 1110–1120. 34083787 10.1038/s41593-021-00865-x

[R15] Dubreuil Alexis, Valente Adrian, Beiran Manuel, Mastrogiuseppe Francesca, and Ostojic Srdjan. 2022. “The Role of Population Structure in Computations through Neural Dynamics.” Nature Neuroscience 25 (6): 783–794. 35668174 10.1038/s41593-022-01088-4 PMC9284159

[R16] Durka Piotr J., Zygierewicz Jarosław, Klekowicz Hubert, Ginter Józef, and Blinowska Katarzyna J.. 2004. “On the Statistical Significance of Event-Related EEG Desynchronization and Synchronization in the Time-Frequency Plane.” IEEE Transactions on Bio-Medical Engineering 51 (7): 1167–1175. 15248533 10.1109/TBME.2004.827341

[R17] Durstewitz Daniel, Vittoz Nicole M., Floresco Stan B., and Seamans Jeremy K.. 2010. “Abrupt Transitions between Prefrontal Neural Ensemble States Accompany Behavioral Transitions during Rule Learning.” Neuron 66 (3): 438–448. 20471356 10.1016/j.neuron.2010.03.029

[R18] Eichenbaum Howard. 2017. “Prefrontal-Hippocampal Interactions in Episodic Memory.” Nature Reviews. Neuroscience 18 (9): 547–558. 28655882 10.1038/nrn.2017.74

[R19] Elston Thomas W., and Wallis Joni D.. 2025. “Context-Dependent Decision-Making in the Primate Hippocampal-Prefrontal Circuit.” Nature Neuroscience 28 (2): 374–382. 39762657 10.1038/s41593-024-01839-5 PMC11802454

[R20] Epstein Russell A., Patai Eva Zita, Julian Joshua B., and Spiers Hugo J.. 2017. “The Cognitive Map in Humans: Spatial Navigation and beyond.” Nature Neuroscience 20 (11): 1504–1513. 29073650 10.1038/nn.4656 PMC6028313

[R21] Euston David R., Gruber Aaron J., and McNaughton Bruce L.. 2012. “The Role of Medial Prefrontal Cortex in Memory and Decision Making.” Neuron 76 (6): 1057–1070. 23259943 10.1016/j.neuron.2012.12.002 PMC3562704

[R22] Flesch Timo, Juechems Keno, Dumbalska Tsvetomira, Saxe Andrew, and Summerfield Christopher. 2022. “Orthogonal Representations for Robust Context-Dependent Task Performance in Brains and Neural Networks.” Neuron 110 (7): 1258–1270.e11. 35085492 10.1016/j.neuron.2022.01.005 PMC8992799

[R23] Franceschini Alessandra, Jin Michelle, Chen Claire W., Silvestri Ludovico, Mastrodonato Alessia, and Denny Christine Ann. 2025. “Brain-Wide Immunolabeling and Tissue Clearing Applications for Engram Research.” Neurobiology of Learning and Memory 218 (108032): 108032. 39922482 10.1016/j.nlm.2025.108032 PMC13202242

[R24] Garner Aleena R., Rowland David C., Hwang Sang Youl, 2012. “Generation of a Synthetic Memory Trace.” Science (New York, N.Y.) 335 (6075): 1513–1516. 22442487 10.1126/science.1214985 PMC3956300

[R25] Guenthner Casey J., Miyamichi Kazunari, Yang Helen H., Craig Heller H., and Luo Liqun. 2013. “Permanent Genetic Access to Transiently Active Neurons via TRAP: Targeted Recombination in Active Populations.” Neuron 79 (6): 1257. 10.1016/j.neuron.2013.03.025 PMC3782391 23764283

[R26] Guskjolen Axel, and Cembrowski Mark S.. 2023. “Engram Neurons: Encoding, Consolidation, Retrieval, and Forgetting of Memory.” Molecular Psychiatry 28 (8): 3207–3219. 37369721 10.1038/s41380-023-02137-5 PMC10618102

[R27] Guskjolen Axel, Kenney Justin W., Parra uan de la, Amy Yeung Bi-Ru, Josselyn Sheena A., and Frankland Paul W.. 2018. “Recovery of ‘Lost’ Infant Memories in Mice.” Current Biology 28 (14): 2283–2290.e3. 29983316 10.1016/j.cub.2018.05.059

[R28] Guzowski John F., Setlow Barry, Wagner Edward K., and McGaugh James L.. 2001. “Experience-Dependent Gene Expression in the Rat Hippocampus after Spatial Learning: A Comparison of the Immediate-Early genesArc, c-Fos, and zif268.” Journal of Neuroscience 21 (14): 5089–5098. 11438584 10.1523/JNEUROSCI.21-14-05089.2001 PMC6762831

[R29] Hanks Timothy D., Kopec Charles D., Brunton Bingni W., Duan Chunyu A., Erlich Jeffrey C., and Brody Carlos D.. 2015. “Distinct Relationships of Parietal and Prefrontal Cortices to Evidence Accumulation.” Nature 520 (7546): 220–223. 25600270 10.1038/nature14066 PMC4835184

[R30] Hirsh Richard. 1974. “The Hippocampus and Contextual Retrieval of Information from Memory: A Theory.” Behavioral Biology 12 (4): 421–444. 4217626 10.1016/s0091-6773(74)92231-7

[R31] Hok V., Save E., Lenck-Santini P. P., and Poucet B.. 2005. “Coding for Spatial Goals in the Prelimbic/infralimbic Area of the Rat Frontal Cortex.” Proceedings of the National Academy of Sciences of the United States of America 102 (12): 4602–4607. 15761059 10.1073/pnas.0407332102 PMC555486

[R32] Hoover Walter B., and Vertes Robert P.. 2007. “Anatomical Analysis of Afferent Projections to the Medial Prefrontal Cortex in the Rat.” Brain Structure & Function 212 (2): 149–179. 17717690 10.1007/s00429-007-0150-4

[R33] Inagaki Hidehiko K., Fontolan Lorenzo, Romani Sandro, and Svoboda Karel. 2019. “Discrete Attractor Dynamics Underlies Persistent Activity in the Frontal Cortex.” Nature 566 (7743): 212–217. 30728503 10.1038/s41586-019-0919-7

[R34] Jadhav Shantanu P., Rothschild Gideon, Roumis Demetris K., and Frank Loren M.. 2016. “Coordinated Excitation and Inhibition of Prefrontal Ensembles during Awake Hippocampal Sharp-Wave Ripple Events.” Neuron 90 (1): 113–127. 26971950 10.1016/j.neuron.2016.02.010 PMC4824654

[R35] Josselyn Sheena A., and Tonegawa Susumu. 2020. “Memory Engrams: Recalling the Past and Imagining the Future.” Science (New York, N.Y.) 367 (6473): eaaw4325. 31896692 10.1126/science.aaw4325 PMC7577560

[R36] Jung M. W., Qin Y., McNaughton B. L., and Barnes C. A.. 1998. “Firing Characteristics of Deep Layer Neurons in Prefrontal Cortex in Rats Performing Spatial Working Memory Tasks.” Cerebral Cortex (New York, N.Y.: 1991) 8 (5): 437–450. 9722087 10.1093/cercor/8.5.437

[R37] Kaefer Karola, Nardin Michele, Blahna Karel, and Csicsvari Jozsef. 2020. “Replay of Behavioral Sequences in the Medial Prefrontal Cortex during Rule Switching.” Neuron 106 (1): 154–165.e6. 32032512 10.1016/j.neuron.2020.01.015

[R38] Langdon Christopher, and Engel Tatiana A.. 2025. “Latent Circuit Inference from Heterogeneous Neural Responses during Cognitive Tasks.” Nature Neuroscience 28 (3): 665–675. 39930096 10.1038/s41593-025-01869-7 PMC11893458

[R39] Lee Gregory, Gommers Ralf, Waselewski Filip, Wohlfahrt Kai, and O’Leary Aaron. 2019. “PyWavelets: A Python Package for Wavelet Analysis.” Journal of Open Source Software 4 (36): 1237.

[R40] Li Nuo, Daie Kayvon, Svoboda Karel, and Druckmann Shaul. 2016. “Robust Neuronal Dynamics in Premotor Cortex during Motor Planning.” Nature 532 (7600): 459–464. 27074502 10.1038/nature17643 PMC5081260

[R41] Liu Xu, Ramirez Steve, Pang Petti T., 2012. “Optogenetic Stimulation of a Hippocampal Engram Activates Fear Memory Recall.” Nature 484 (7394): 381–385. 22441246 10.1038/nature11028 PMC3331914

[R42] Luo Thomas Zhihao, Bondy Adrian Gopnik, Gupta Diksha, Elliott Verity Alexander, Kopec Charles D., and Brody Carlos D.. 2020. “An Approach for Long-Term, Multi-Probe Neuropixels Recordings in Unrestrained Rats.” eLife 9 (e59716): e59716. 33089778 10.7554/eLife.59716 PMC7721443

[R43] Mante Valerio, Sussillo David, Shenoy Krishna V., and Newsome William T.. 2013. “Context-Dependent Computation by Recurrent Dynamics in Prefrontal Cortex.” Nature (England) 503 (7474): 78–84. 10.1038/nature12742 PMC4121670 24201281

[R44] Maren Stephen, Luan Phan K., and Liberzon Israel. 2013. “The Contextual Brain: Implications for Fear Conditioning, Extinction and Psychopathology.” Nature Reviews. Neuroscience 14 (6): 417–428. 23635870 10.1038/nrn3492 PMC5072129

[R45] Maris Eric, and Oostenveld Robert. 2007. “Nonparametric Statistical Testing of EEG- and MEG-Data.” Journal of Neuroscience Methods 164 (1): 177–190. 17517438 10.1016/j.jneumeth.2007.03.024

[R46] Miller E. K., and Cohen J. D.. 2001. “An Integrative Theory of Prefrontal Cortex Function.” Annual Review of Neuroscience 24 (1): 167–202. 10.1146/annurev.neuro.24.1.167 11283309

[R47] Milner Brenda. 1963. “Effects of Different Brain Lesions on Card Sorting: The Role of the Frontal Lobes.” Archives of Neurology 9 (1): 90.

[R48] Monsell Stephen. 2003. “Task Switching.” Trends in Cognitive Sciences 7 (3): 134–140. 12639695 10.1016/s1364-6613(03)00028-7

[R49] Monsell S., Yeung N., and Azuma R.. 2000. “Reconfiguration of Task-Set: Is It Easier to Switch to the Weaker Task?” Psychological Research 63 (3–4): 250–264. 11004879 10.1007/s004269900005

[R50] Munoz Douglas P., and Everling Stefan. 2004. “Look Away: The Anti-Saccade Task and the Voluntary Control of Eye Movement.” Nature Reviews. Neuroscience 5 (3): 218–228. 14976521 10.1038/nrn1345

[R51] Norman Jacob F., Rahsepar Bahar, Vena Anna, 2025. “Reactivation of Memory-Associated Neurons Induces Downstream Suppression of Competing Neuronal Populations.” Proceedings of the National Academy of Sciences of the United States of America 122 (14): e2410101122. 40168126 10.1073/pnas.2410101122 PMC12002025

[R52] O’Keefe John, and Nadel Lynn. 1978. The Hippocampus as a Cognitive Map. Oxford University Press.

[R53] Pagan Marino, Tang Vincent D., Aoi Mikio C., 2025. “Individual Variability of Neural Computations Underlying Flexible Decisions.” Nature 639 (8054): 421–429. 39608399 10.1038/s41586-024-08433-6 PMC11903320

[R54] Pavlova Ina P., Shipley Shannon C., Lanio Marcos, Hen René, and Denny Christine A.. 2018. “Optimization of Immunolabeling and Clearing Techniques for Indelibly Labeled Memory Traces.” Hippocampus 28 (7): 523–535. 29663578 10.1002/hipo.22951 PMC6021204

[R55] Pfurtscheller G., and Lopes da Silva F. H.. 1999. “Event-Related EEG/MEG Synchronization and Desynchronization: Basic Principles.” Clinical Neurophysiology: Official Journal of the International Federation of Clinical Neurophysiology 110 (11): 1842–1857. 10576479 10.1016/s1388-2457(99)00141-8

[R56] Pinto Lucas, Koay Sue A., Engelhard Ben, 2018. “An Accumulation-of-Evidence Task Using Visual Pulses for Mice Navigating in Virtual Reality.” Frontiers in Behavioral Neuroscience 12: 36. 29559900 10.3389/fnbeh.2018.00036 PMC5845651

[R57] Place Ryan, Farovik Anja, Brockmann Marco, and Eichenbaum Howard. 2016. “Bidirectional Prefrontal-Hippocampal Interactions Support Context-Guided Memory.” Nature Neuroscience 19 (8): 992–994. 27322417 10.1038/nn.4327 PMC4961615

[R58] Rahsepar Bahar, Norman Jacob F., Noueihed Jad, 2023. “Theta-Phase-Specific Modulation of Dentate Gyrus Memory Neurons.” eLife 12 (e82697): e82697. 37401757 10.7554/eLife.82697 PMC10361715

[R59] Ramirez Steve, Liu Xu, Lin Pei-Ann, 2013. “Creating a False Memory in the Hippocampus.” Science (New York, N.Y.) 341 (6144): 387–391. 23888038 10.1126/science.1239073

[R60] Redondo Roger L., Kim Joshua, Arons Autumn L., Ramirez Steve, Liu Xu, and Tonegawa Susumu. 2014. “Bidirectional Switch of the Valence Associated with a Hippocampal Contextual Memory Engram.” Nature 513 (7518): 426–430. 25162525 10.1038/nature13725 PMC4169316

[R61] Renier Nicolas, Adams Eliza L., Kirst Christoph, 2016. “Mapping of Brain Activity by Automated Volume Analysis of Immediate Early Genes.” Cell 165 (7): 1789–1802. 27238021 10.1016/j.cell.2016.05.007 PMC4912438

[R62] Renier Nicolas, Wu Zhuhao, Simon David J., Yang Jing, Ariel Pablo, and Tessier-Lavigne Marc. 2014. “iDISCO: A Simple, Rapid Method to Immunolabel Large Tissue Samples for Volume Imaging.” Cell 159 (4): 896–910. 25417164 10.1016/j.cell.2014.10.010

[R63] Rigotti Mattia, Barak Omri, Warden Melissa R., 2013. “The Importance of Mixed Selectivity in Complex Cognitive Tasks.” Nature 497 (7451): 585–590. 23685452 10.1038/nature12160 PMC4412347

[R64] Roy Dheeraj S., Park Young-Gyun, Kim Minyoung E., 2022. “Brain-Wide Mapping Reveals That Engrams for a Single Memory Are Distributed across Multiple Brain Regions.” Nature Communications 13 (1): 1799. 10.1038/s41467-022-29384-4 PMC8980018 35379803

[R65] Ryan Tomás J., Roy Dheeraj S., Pignatelli Michele, Arons Autumn, and Tonegawa Susumu. 2015. “Engram Cells Retain Memory under Retrograde Amnesia.” Science (New York, N.Y.) 348 (6238): 1007–1013. 26023136 10.1126/science.aaa5542 PMC5583719

[R66] Sakai Katsuyuki. 2008. “Task Set and Prefrontal Cortex.” Annual Review of Neuroscience 31 (1): 219–245. 10.1146/annurev.neuro.31.060407.125642 18558854

[R67] Sauer Jonas-Frederic, Folschweiller Shani, and Bartos Marlene. 2022. “Topographically Organized Representation of Space and Context in the Medial Prefrontal Cortex.” Proceedings of the National Academy of Sciences of the United States of America 119 (6): e2117300119. 35121665 10.1073/pnas.2117300119 PMC8833199

[R68] Siegel Markus, Buschman Timothy J., and Miller Earl K.. 2015. “Cortical Information Flow during Flexible Sensorimotor Decisions.” Science (New York, N.Y.) 348 (6241): 1352–1355. 26089513 10.1126/science.aab0551 PMC4721574

[R69] Smith David M., and Mizumori Sheri J. Y.. 2006. “Hippocampal Place Cells, Context, and Episodic Memory.” Hippocampus 16 (9): 716–729. 16897724 10.1002/hipo.20208

[R70] Spellman Timothy, Rigotti Mattia, Ahmari Susanne E., Fusi Stefano, Gogos Joseph A., and Gordon Joshua A.. 2015. “Hippocampal-Prefrontal Input Supports Spatial Encoding in Working Memory.” Nature 522 (7556): 309–314. 26053122 10.1038/nature14445 PMC4505751

[R71] Suthard Rebecca L., Senne Ryan A., Buzharsky Michelle D., Diep Anh H., Pyo Angela Y., and Ramirez Steve. 2024. “Engram Reactivation Mimics Cellular Signatures of Fear.” Cell Reports 43 (3): 113850. 38401120 10.1016/j.celrep.2024.113850

[R72] Tanaka Kazumasa Z., Pevzner Aleksandr, Hamidi Anahita B., Nakazawa Yuki, Graham Jalina, and Wiltgen Brian J.. 2014. “Cortical Representations Are Reinstated by the Hippocampus during Memory Retrieval.” Neuron 84 (2): 347–354. 25308331 10.1016/j.neuron.2014.09.037

[R73] Tang Wenbo, Shin Justin D., and Jadhav Shantanu P.. 2021. “Multiple Time-Scales of Decision-Making in the Hippocampus and Prefrontal Cortex.” eLife 10: e66227. 33683201 10.7554/eLife.66227 PMC7993991

[R74] Tavares Lucas C. S., and Tort Adriano B. L.. 2022. “Hippocampal–prefrontal Interactions during Spatial D Ecision-making.” Hippocampus 32 (1): 38–54. 34843143 10.1002/hipo.23394

[R75] Teyler T. J., and DiScenna P.. 1986. “The Hippocampal Memory Indexing Theory.” Behavioral Neuroscience 100 (2): 147–154. 3008780 10.1037//0735-7044.100.2.147

[R76] Thierry Anne-Marie, Gioanni Yves, Dégénétais Eric, and Glowinski Jacques. 2000. “Hippocampo-Prefrontal Cortex Pathway: Anatomical and Electrophysiological Characteristics.” Hippocampus 10 (4): 411–419. 10985280 10.1002/1098-1063(2000)10:4<411::AID-HIPO7>3.0.CO;2-A

[R77] Tyson Adam L., Vélez-Fort Mateo, Rousseau Charly V., 2022. “Accurate Determination of Marker Location within Whole-Brain Microscopy Images.” Scientific Reports 12 (1): 867. 35042882 10.1038/s41598-021-04676-9 PMC8766598

[R78] Virtanen Pauli, Gommers Ralf, Oliphant Travis E., 2020. “SciPy 1.0: Fundamental Algorithms for Scientific Computing in Python.” Nature Methods 17 (3): 261–272. 32015543 10.1038/s41592-019-0686-2 PMC7056644

[R79] Weilbächer Regina A., and Gluth Sebastian. 2016. “The Interplay of Hippocampus and Ventromedial Prefrontal Cortex in Memory-Based Decision Making.” Brain Sciences 7 (1): 4. 28036071 10.3390/brainsci7010004 PMC5297293

[R80] Wikenheiser Andrew M., Marrero-Garcia Yasmin, and Schoenbaum Geoffrey. 2017. “Suppression of Ventral Hippocampal Output Impairs Integrated Orbitofrontal Encoding of Task Structure.” Neuron 95 (5): 1197–1207. 28823726 10.1016/j.neuron.2017.08.003 PMC5637553

[R81] Wimmer Ralf D., Ian Schmitt L., Davidson Thomas J., Nakajima Miho, Deisseroth Karl, and Halassa Michael M.. 2015. “Thalamic Control of Sensory Selection in Divided Attention.” Nature 526 (7575): 705–709. 26503050 10.1038/nature15398 PMC4626291

[R82] Wu Zheng, Litwin-Kumar Ashok, Shamash Philip, Taylor Alexei, Axel Richard, and Shadlen Michael N.. 2020. “Context-Dependent Decision Making in a Premotor Circuit.” Neuron 106 (2): 316–328. 32105611 10.1016/j.neuron.2020.01.034

[R83] Ye Xiaojing, Kapeller-Libermann Dana, Travaglia Alessio, Carmen Inda M., and Alberini Cristina M.. 2017. “Direct Dorsal Hippocampal-Prelimbic Cortex Connections Strengthen Fear Memories.” Nature Neuroscience 20 (1): 52–61. 27869801 10.1038/nn.4443 PMC5191950

[R84] Zielinski Mark C., Shin Justin D., and Jadhav Shantanu P.. 2019. “Coherent Coding of Spatial Position Mediated by Theta Oscillations in the Hippocampus and Prefrontal Cortex.” The Journal of Neuroscience: The Official Journal of the Society for Neuroscience 39 (23): 4550–4565. 30940717 10.1523/JNEUROSCI.0106-19.2019 PMC6554624

[R85] Zimmerman Christopher A., Bolkan Scott S., Pan-Vazquez Alejandro, 2025. “A Neural Mechanism for Learning from Delayed Postingestive Feedback.” Nature 642 (8068): 700–709. 40175547 10.1038/s41586-025-08828-z PMC12176619

